# Dual renin-angiotensin system inhibition for prevention of renal and cardiovascular events: do the latest trials challenge existing evidence?

**DOI:** 10.1186/1475-2840-12-108

**Published:** 2013-07-19

**Authors:** Samir G Mallat

**Affiliations:** 1 Department of Internal Medicine, American University of Beirut Medical Center (AUBMC), PO Box 11-0236, Riad-El-Solh, Beirut 1107 2020, Lebanon

**Keywords:** Angiotensin-converting enzyme (ACE) inhibitors, Angiotensin II receptor blockers, Blood pressure, Cardiovascular disease, Dual renin-angiotensin system inhibition, Direct renin inhibitors, Outcomes, Renal disease

## Abstract

Circulatory and tissue renin-angiotensin systems (RAS) play a central role in cardiovascular (CV) and renal pathophysiology, making RAS inhibition a logical therapeutic approach in the prevention of CV and renal disease in patients with hypertension. The cardio- and renoprotective effects observed with angiotensin-converting enzyme (ACE) inhibitors or angiotensin II receptor blockers (ARBs) monotherapy, together with the availability of a direct renin inhibitor (DRI), led to the investigation of the potential benefits of dual RAS inhibition. In small studies, ARB and ACE inhibitor combinations were shown to be beneficial in patients with CV or renal disease, with improvement in surrogate markers. However, in larger outcome trials, involving combinations of ACE inhibitors, ARBs or DRIs, dual RAS inhibition did not show reduction in mortality in patients with diabetes, heart failure, coronary heart disease or after myocardial infarction, and was in fact, associated with increased harm. A recent meta-analysis of all major trials conducted over the past 22 years involving dual RAS inhibition has clearly shown that the risk-benefit ratio argues against the use of dual RAS inhibition. Hence, the recent evidence clearly advocates against the use of dual RAS inhibition, and single RAS inhibition appears to be the most suitable approach to controlling blood pressure and improving patient outcomes.

## Introduction

The central role of the renin-angiotensin system (RAS) in the regulation of blood pressure (BP) has been recognized for many years. The discovery of tissue-based angiotensin II production has resulted in the concept of a local RAS that is independent of the circulating RAS.

**Figure 1:**
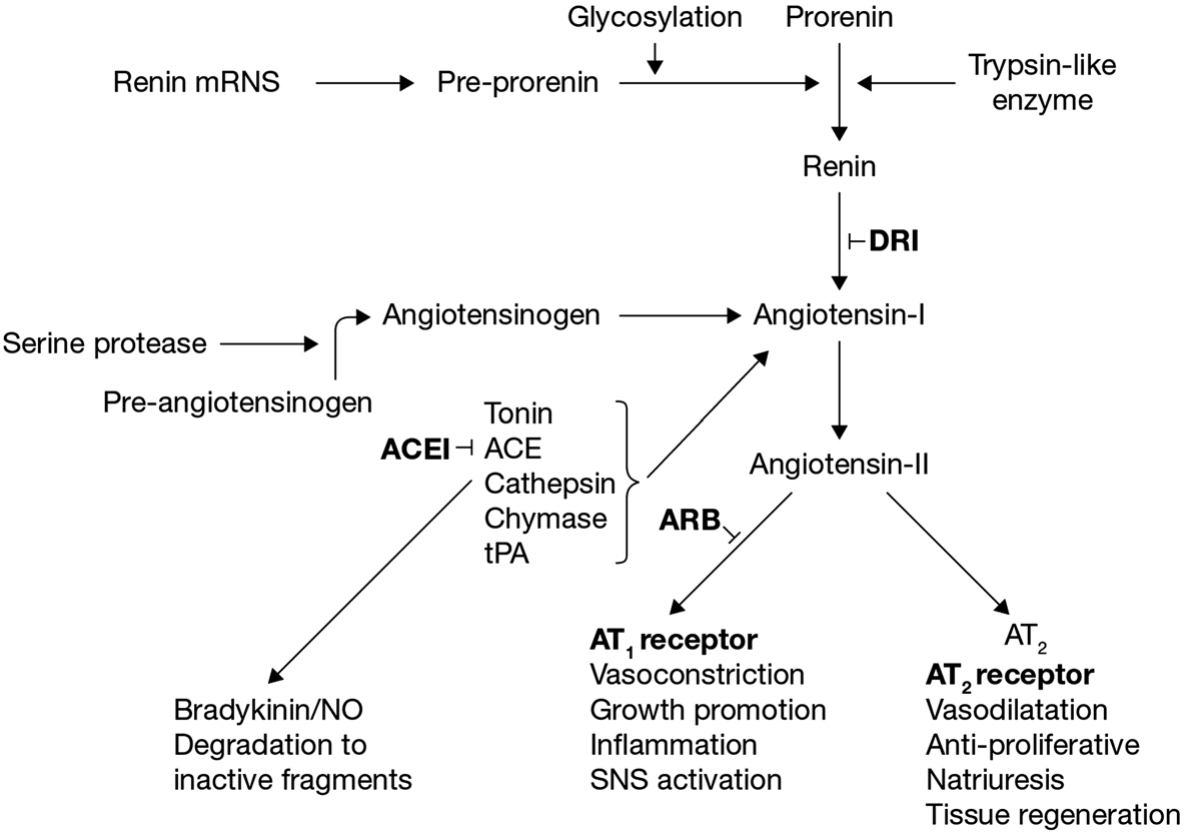
**Schematic representation of the RAS.** ACE, angiotensin-converting enzyme; ARB, angiotensin II receptor blocker; AT_1_, angiotensin II type 1 receptor; AT_2_, angiotensin II type 2 receptor; DRI, direct renin inhibitor; NO, nitric oxide; PRA, plasma renin activity; PRC, plasma renin concentration; RAS, renin-angiotensin system; SNS, sympathetic nervous system; tPA, tissue plasminogen activator. Adapted from Farsang 2011 [9].

RAS activation following the release of renin by the kidney results in a cascade of physiological events (Figure 1). Renin catalyzes the formation of angiotensin I, which is then converted to angiotensin II by angiotensin-converting enzyme (ACE), resulting in activation of the angiotensin II receptors and its deleterious effect on renal vasculature, resulting in chronic kidney disease (CKD) [1-6]. The progressive development of cardiovascular (CV) disease (CVD) resulting from pathophysiological changes mediated by angiotensin II in the presence of risk factors is well established [2] and local activation of RAS in the vascular walls is thought to contribute to atherosclerosis [5]*.* Furthermore, intrarenal RAS is often inappropriately activated in diabetes and is thought to predispose these patients to nephropathy [7,8]. RAS inhibition (both circulatory and intrarenal) is therefore a key therapeutic approach to slow progression of CKD and to reduce CV risk through both BP-dependent and independent mechanisms.

All three classes of available RAS inhibitors (ACE inhibitors, angiotensin receptor blockers [ARBs] and direct renin inhibitors [DRIs]) interrupt the normal angiotensin II feedback suppression of renin secretion from the kidneys [10]. In the past two decades, landmark trials have shown that early aggressive lowering of BP and inhibition of the RAS improves outcomes for patients with renal disease or CVD [11-15]. ACE inhibitors and ARBs reduce proteinuria, slow progression of CKD and lower morbidity and mortality rates in patients at high CVD risk, and in patients already displaying evidence of target organ damage (TOD) such as myocardial infarction (MI), heart failure (HF), stable coronary heart disease (CHD) with or without left ventricular dysfunction (LVD), and reduce mortality and reinfarction rates in patients with LVD or HF after MI [12-32]. Evidence from large outcome trials such as the ONgoing Telmisartan Alone and in combination with Ramipril Global Endpoint Trial (ONTARGET^®^) suggests that ARBs like telmisartan have additional CV benefits beyond BP lowering [33]. Outcomes with ARB monotherapy in post-MI patients are similar to those achieved with high doses of an ACE inhibitor [28,34]. ACE inhibitors and ARBs are widely acknowledged to confer additional renoprotective benefits beyond the effects of BP control alone [35] (Table 1). ARBs are also known to activate peroxisome proliferator-activated receptor gamma (PPAR-γ), however, only telmisartan exhibits increased PPAR-γ activity at therapeutic dosages [36,37]. PPAR-γ enhances insulin sensitivity, has positive effects on lipid metabolism, endothelium, oxidative stress and vascular inflammation, and its anti-inflammatory, antiatherogenic and antihypertensive effects are considered to exert CV protective effects [38,39]. Initial data suggest that, as with ARBs and ACE inhibitors, aliskiren, an oral DRI, may protect against TOD [40-42].

**Table 1:** Renoprotective effects of ACE inhibitors and ARBs (large [n ~ or > 100] randomized controlled studies)

**Study population**	**Treatment arms**	**Main findings: statistically significant renoprotective effect of ACE inhibitor/ARB versus comparator**
**ACE inhibitor**		
IDDM patients with macroalbuminuria [17]	Captopril or placebo	48% RR in doubling of serum creatinine; 50% RR of the combined endpoint (death, dialysis and transplantation)
T2DM patients with albuminuria [43]	Enalapril or placebo	1% versus 13% decline in kidney function
Nondiabetic patients with proteinuria [44]	Ramipril or placebo	Lower decline in GFR (0.53 ml/min versus 0.88 ml/min); RR in doubling of baseline creatinine or ESRD
T2DM patients [45]	Ramipril or placebo	24% risk reduction in overt nephropathy
T2DM patients [46]	Enalapril or nifedipine	Greater reduction in albuminuria; 23.8% patients (versus 15.4%) reverted to normoalbuminuria; 19.1% patients (versus 30.8%) developed macroalbuminuria
**ARB**		
T2DM patients with nephropathy [13]	Losartan or placebo	16% RR in doubling of serum creatinine concentration; 28% RR in ESRD; significant reduction in proteinuria versus placebo
Hypertensive T2DM with nephropathy [11]	Irbesartan or amlodipine or placebo	RR of doubling serum creatinine and of ESRD
T2DM patients with nephropathy [15]	Irbesartan or placebo	Reduction in the onset of diabetic nephropathy compared with placebo
T2DM patients [47]	Telmisartan or placebo	RR in progression to overt nephropathy; reduced UACR; increased microalbuminuria remission
T2DM patients with hypertension [48]	Telmisartan or ramipril	Both telmisartan and ramipril increase NO activity of renal endothelium which may preserve renal function
CVD and T2DM patients [49]	Telmisartan or placebo	Prevented increase in albuminuria (32% increase versus 63% increase); however, telmisartan was associated with a greater doubling of serum creatinine and decrease in estimated GFR, although there was no difference in terms of renal outcome
T2DM patients [50]	Valsartan or amlodipine	Greater reduction in urine albumin excretion (44% versus 8%)

*ACE,* angiotensin-converting enzyme; *ARB,* angiotensin II receptor blocker; *CVD,* cardiovascular disease; *ESRD,* end-stage renal disease; *GFR,* glomerular filtration rate; *IDDM,* insulin-dependent diabetes mellitus; *NO,* nitric oxide; *RR*, risk reduction; *T2DM*, Type 2 diabetes mellitus; *UACR*, urinary albumin-creatinine ratio.

Dual RAS inhibition was theorized to result in better RAS inhibition, giving rise to greater benefit on BP lowering and cardiorenal outcomes. Early studies on dual RAS inhibition with ACE inhibitors and ARBs have shown greater reduction in BP with the combination [51], but benefits on surrogate endpoints and outcomes have not been consistent [22,28,52-61]. The ONTARGET^®^ study, the largest trial of dual RAS inhibition in high-risk patients (those with CVD or diabetes but not HF), in which patients were randomized to receive either telmisartan or ramipril, or a combination of the two agents, found no evidence to support the use of dual RAS inhibition in these patients [33,62]. This article reviews the recent evidence, including those from large outcome trials (Table 2), for the efficacy of dual RAS inhibition in patients at a high risk of CVD with multiple co-morbidities such as LVD, HF, CKD and TOD.

**Table 2:** Overview of large outcome trials investigating dual RAS inhibition

**Study name or author**	**Study population**	**ACE inhibitor**	**ARB**	**DRI**	**Impact of combined therapy versus monotherapy**
ONTARGET^®^[33,63]	Patients at high risk of CV events	Ramipril	Telmisartan		No improvement in CV outcomes; increased incidence of renal events due to acute renal failure caused by concomitant diseases (tumour, pneumonia, severe diabetes and others)
ALOFT [64]	HF	Standard therapy		Aliskiren	Addition of aliskiren to standard treatment reduced NT-proBNP. Combined therapy had no effect on UACR
ALLAY [65]	Hypertensive patient with LVH		Losartan	Aliskiren	No additional benefit over and above monotherapy
ALTITUDE [66]	T2DM patients	Standard therapy		Aliskiren	Increased incidence of AEs in the combination arm (including non-fatal stroke, renal dysfunction, hyperkalaemia and hypotension)
AVOID [12]	T2DM patients		Losartan	Aliskiren	Significantly reduced UACR versus losartan monotherapy
ASPIRE [67]	Patients following acute MI	Standard therapy		Aliskiren	Combined therapy did not further attenuate left-ventricular remodelling
ASTRONAUT [68]	Haemodynamically stable, hospitalizations for heart failure patients	Standard therapy		Aliskiren	No reduction in CV death or HF rehospitalization at 6 months or 12 months after discharge
VA NEPHRON-D [61,69]	Patients with diabetes and overt proteinuria	Lisinopril	Losartan		Terminated early due to greater number of observed acute kidney injury events and hyperkalaemia in the combination group

*ACE,* angiotensin-converting enzyme; *AE,* adverse event; *ARB,* angiotensin II receptor blocker; *ALLAY,* ALiskiren Left ventricular Assessment of hypertrophY; *ALOFT,* ALiskiren Observation of heart Failure Treatment; *ALTITUDE,* ALiskiren Trial in Type 2 diabetes Using carDio-renal Endpoints; *ASPIRE,* Aliskiren Study in Post-MI Patients to Reduce rEmodeling; *ASTRONAUT,* The AliSkiren TRial ON Acute heart failure oUTcomes; *AVOID,* Aliskiren in the eValuation of prOteinuria in Diabetes; *CV,* cardiovascular; *HF,* heart failure; *LVH,* left ventricular hypertrophy; *MI,* myocardial infarction; *NT-proBNP,* N-terminal prohormone of brain natriuretic peptide; *ONTARGET*^*®*^*,* ONgoing Telmisartan Alone and in combination with Ramipril Global Endpoint Trial; *RAS,* renin-angiotensin system; *T2DM,* Type 2 diabetes mellitus; *UACR,* urine albumin-creatinine ratio; *VA NEPHRON-D,* Combination Angiotensin Receptor Blocker and Angiotensin-Converting Enzyme Inhibitor for Treatment of Diabetic Nephropathy.

Standard therapy: ALOFT and ASPIRE–ACE inhibitor (or ARB) and β-blocker; ALTITUDE–ACE inhibitor or ARB; ASTRONAUT–included diuretics, ACE inhibitors or ARBs, β-blockers and aldosterone blocking agents, unless contraindicated.

### Study selection

The PubMed database was systematically searched for English language articles published during the period May 2008 to May 2013, reporting results of trials comparing dual blockers of the RAS with monotherapy. The search terms used were “angiotensin-converting enzyme inhibitor”, “angiotensin receptor blocker”, “cardiovascular disease”, “chronic kidney disease”, “diabetes”, “direct renin inhibitor”, “dual RAS blockade”, “heart failure”, “myocardial infarction”. The reference lists of the articles retrieved by the electronic search also were searched for other potentially eligible articles. This review also was supplemented with publications of landmark studies on single RAS inhibition that fell outside the search criteria.

### High-risk patients with LVD or HF

A series of landmark trials with ACE inhibitors in patients with LVD or HF such as VALsartan in Acute myocardial INfarction (VALIANT), Valsartan in Heart Failure Trial (Val-HeFT) and others have reported reductions in CV mortality and morbidity [22,23,25,27,28,31,32]. Evidence for efficacy of ARB monotherapy in this patient group is provided by the Evaluation of Losartan In The Elderly (ELITE) I and II studies [29,30], Candesartan in Heart failure Assessment of Reduction in mortality and Morbidity (CHARM) [53,54,58,59], ONTARGET^®^[33] and Telmisartan Randomized AssessmeNt Study in ACE-I iNtolerant subjects with cardiovascular Disease (TRANSCEND^®^) [70].

There is limited, but conflicting, evidence on the effects of dual RAS inhibition on mortality in patients with LVD or HF. The prevalence of left ventricular hypertrophy (LVH) was reduced by dual RAS inhibition in ONTARGET^®^ (odds ratio, 0.93; 95% confidence interval (CI), 0.84 to 1.02; p = 0.12 compared with ramipril). In a pilot study in 24 treatment-naive hypertensive patients with left ventricular (LV) concentric hypertrophy randomized to ramipril plus candesartan or ramipril plus lercanidipine, the decrease of LV mass and the improvement of diastolic function were significantly greater with ramipril plus candesartan, suggesting a greater antiremodelling effect [71]. A benefit of dual RAS inhibition, of telmisartan and ACE inhibitors was apparent in a small study (n = 332) in patients with end-stage renal disease (ESRD) in addition to HF and impaired left ventricular ejection fraction (LVEF). The dual therapy significantly improved the three primary outcomes of all-cause mortality (35.1% versus 54.4%; p < 0.001), CV mortality (30.3% versus 43.7%; p < 0.001) and hospital admission for chronic HF (33.9% versus 55.1%; p < 0.0001) compared with placebo [72]. In post-acute MI / percutaneous coronary intervention Japanese patients, treatment with half-dose combination of valsartan and trandolapril was observed to suppress LV remodelling more effectively than trandolapril alone [73]. The ALiskiren Left ventricular Assessment of hypertrophY (ALLAY) trial randomized 465 hypertensive overweight patients with LVH to aliskiren 300 mg, losartan 100 mg or the combination for 9 months. The combination of aliskiren plus losartan was not significantly different from losartan for the primary outcome of reduction in LV mass [65]. In a subset of 136 patients who had plasma aldosterone concentration measured at baseline and study end, aliskiren, either alone or in combination, resulted in a significantly greater reduction from baseline to 9 months in plasma aldosterone than losartan alone, and the suppression of aldosterone was associated with reduction of LVH, independently of the change in systolic blood pressure (SBP) [74].

In the ALiskiren Observation of heart Failure Treatment (ALOFT) study, patients with stable HF, and treated with an ACE inhibitor (or ARB) and β-blocker were randomized to once-daily, double-blind treatment with aliskiren 150 mg or placebo. After 3 months of treatment, plasma N-terminal prohormone of brain natriuretic peptide (NT-proBNP) was significantly reduced with aliskiren, suggesting favourable neurohumoral effects in heart failure [64]. These beneficial changes in neurohumoral biomarkers were observed regardless of concomitant mineralocorticoid treatment received by 33% of patients. The incidence of pre-specified adverse events (AEs) of renal dysfunction, symptomatic hypotension and hyperkalaemia was low, and not significantly different between aliskiren and placebo, irrespective of whether or not patients were receiving mineralocorticoid treatment [75].

A meta-analysis of trials comparing ACE inhibitors alone or in combination with ARBs in patients with LVD or HF showed an increased risk of developing any AE, hypotension, worsening renal function and hyperkalaemia with combination therapy, suggesting ACE inhibitors should not be routinely used in combination with ARBs in patients with LVD [76]. A later meta-analysis of trials comparing ACE inhibitors with ACE inhibitors plus ARBs in patients with HF showed fewer hospital admissions for HF with combination therapy (with significant heterogeneity between included trials), but no difference for overall mortality, hospitalization for any reason and fatal or nonfatal MI. Also, patients on combination therapy had a higher risk of worsening renal function and symptomatic hypotension, and a higher rate of permanent discontinuation of trial medications [77].

The AliSkiren TRial ON Acute heart failure oUTcomes (ASTRONAUT) was a double-blind, placebo-controlled study in which haemodynamically stable hospitalized HF patients were randomly allocated a median 5 days after admission to receive 150 mg (increased to 300 mg as tolerated) of aliskiren or placebo daily, in addition to standard therapy, which was continued after discharge for a median 11.3 months. At randomization, patients (n = 1639) were receiving diuretics (95.9%), β-blockers (82.5%), ACE inhibitors or ARBs (84.2%), and mineralocorticoid receptor antagonists (57.0%). At 6 and 12 months, addition of aliskiren to standard therapy did not reduce the main outcome measures of CV death or HF rehospitalization. The rates of hyperkalaemia, hypotension and renal impairment / renal failure were higher in the aliskiren group compared with placebo [68]. In the CHARM study, the effects of candesartan in patients with low LVEF receiving ACE inhibitors (CHARM-Added), and patients intolerant to ACE inhibitors (CHARM-Alternative), deaths and hospital admissions for HF were reduced to a greater extent by candesartan than by standard antihypertensive treatment [53,54]. However, in patients with preserved LVF, candesartan did not significantly reduce CV mortality or hospitalizations for HF [54].

### High-risk patients with CKD / Proteinuria

ACE inhibitors and ARBs are widely acknowledged to confer additional renoprotective benefits beyond the effects of BP control alone [35] (Table 1). Inhibition of the RAS slows the progression of renal disease in patients with diabetes, hypertension and albuminuria, but also decreases the risk of CV events [15,17,45,78,79]. Few trials have specifically evaluated the effects of dual RAS inhibition on mortality in patients with CKD. Instead, most studies have considered surrogate endpoints [80].

In the ONTARGET^®^ trial, dual RAS inhibition was associated with a greater risk for the composite outcome of dialysis, doubling of serum creatinine and death (compared with ramipril monotherapy) [63], even though the increase in urine albumin to creatinine ratio (relative to baseline) was less (p < 0.001). Similarly, a post-hoc analysis of the ONTARGET^®^ and TRANSCEND^®^ trials that stratified patients by glomerular filtration rate (GFR) and albuminuria did not support dual RAS inhibition over single RAS inhibition in high vascular risk patients with low GFR or albuminuria [81]. A meta-analysis / metaregression of trials in patients with primary glomerulonephritis showed that the antiproteinuric response to ACE inhibitor plus ARB therapy versus either monotherapy is consistently greater and strictly related to baseline proteinuria, associated with only moderate increase in serum potassium levels and not peculiar to immunoglobulin A nephropathy [82]. In a retrospective analysis of 6-month data from 16 patients with a single kidney and proteinuria, dual RAS blockade with several different ACE inhibitors and ARBs at the maximal dose tolerated by the patient did not affect plasma creatinine levels or creatinine clearance, but also did not reduce proteinuria, suggesting lack of benefit in these patients [83]. In a meta-analysis of 49 randomized trials, which excluded the combination treatment of angiotensin-II receptor blocker and angiotensin-converting-enzyme inhibitor in non-diabetic renal disease (COOPERATE) trial (which was retracted due to serious concerns about the study data), monotherapy with ARBs and ACE inhibitors was reported to delay progression of proteinuria over both the short (1–4 months) and longer (5–12 months) term [84]. Although the data were limited, the combination of the two drugs was considered to reduce proteinuria more than either drug alone [84].

In a small, randomized cross-over study, 22 patients with biopsy-proven immunoglobulin A (IgA) nephropathy and persistent proteinuria despite ACE inhibitor or ARB, were randomized to either oral aliskiren 300 mg/day or placebo for 16 weeks and then crossed over to the other treatment arm after a washout period. After aliskiren treatment, there was a significant reduction in proteinuria in 4 weeks, which remained at a low level throughout the treatment period. There was a significant difference in proteinuria between the aliskiren and placebo groups from 4 to 16 weeks after treatment. After aliskiren treatment, there were modest but statistically significant reductions in estimated GFR (eGFR) and diastolic BP. None of the patients developed severe hyperkalaemia (serum potassium ≥ 6.0 mmol/l) during the study period [85]. In a randomized, open-controlled, cross-over study performed in 18 whites with chronic nondiabetic proteinuric kidney disease, after 8 weeks’ combination treatment with telmisartan 80 mg and aliskiren 300 mg (compared with the combination of telmisartan 80 mg and eplerenone 50 mg and telmisartan 160 mg alone), the urinary excretion of transforming growth factor β-1 was stable, despite a significant increase in plasma renin concentration, and there were no differences in renal function and potassium serum level between the treatments. There were no episodes of hypotension and acute renal impairment. Adverse effects were not reported, suggesting that the combination therapy with telmisartan and aliskiren may be safe in young nondiabetic patients with I-II stage CKD at low vascular risk [86]. In a meta-analysis, combined treatment with an ACE inhibitor plus ARB was found to be more effective than monotherapy with an ACE inhibitor / ARB alone for reducing daily proteinuria in IgA nephropathy, without an increased risk of hyperkalaemia, but no improvement in GFR was observed [87].

In a retrospective cohort study of elderly patients in clinical practice, increased risks of adverse renal outcomes and hyperkalaemia were observed in those on combination therapy with an ACE inhibitor and ARB compared with those on monotherapy [88]. In day-to-day clinical practice, a multimodal strategy (Remission Clinic) of dual RAS inhibition with ACE inhibitors and ARBs up-titrated to maximum tolerated doses, intensified BP control, amelioration of dyslipidaemia by statins, smoking cessation and healthy lifestyle implementation, was found to safely and effectively achieve remission or regression of proteinuria and stabilize kidney function in most CKD patients with heavy proteinuria despite ACE inhibitor therapy, and almost fully prevent progression to ESRD [89]. In a community setting, retrospective cohort-based study, dual therapy with ACE inhibitor and ARB was associated with hyperkalaemia and a decrease in renal function. The absolute risks were especially high among patients with reduced baseline renal function [90].

In an open-label study, 47 patients with advanced CKD were block randomized to 16 weeks of monotherapy with increasing doses of RAS blockade aiming at enalapril 20 mg once daily or candesartan 16 mg once daily, followed by 5 weeks of combination therapy aiming at combined enalapril 20 mg and candesartan 16 mg for 3 weeks. Twenty-one patients (45%) did not tolerate dual blockade due to unacceptable plasma creatinine increase, hypotension, general discomfort or unmanageable hyperkalaemia. Hyperkalaemia was reported in seven (15%) patients [91]. In another open-label study, 67 CKD patients were randomized to 16 weeks of monotherapy with either enalapril or candesartan followed by 8 weeks of dual blockade aiming at a total dose of 16 mg candesartan and 20 mg enalapril once daily. Dual blockade resulted in significant BP-independent reductions in aortic pulse wave volume and in augmentation index compared with monotherapy. Furthermore, pulse pressure amplification was improved and central systolic BP was reduced [92]. In a prospective, 12-month study in patients with nondiabetic proteinuria, with normal or slightly impaired renal function, combination therapy with ramipril and valsartan was more efficacious than either monotherapy in reducing proteinuria and serum creatinine level in the first 3 (proteinuria and serum creatinine) or 6 (serum creatinine) months of treatment [93].

### High-risk patients with diabetes

Diabetes mellitus is considered as a major risk factor for CVD [94], and CVD is the leading cause of death in Type 2 diabetes mellitus (T2DM) patients [95]. Guidelines recommend RAS inhibitors as the first monotherapy for diabetes patients with hypertension [96,97]. Landmark trials such as Action in Diabetes and Vascular Disease: Preterax and Diamicron MR Controlled Evaluation (ADVANCE) [98], Action to Control Cardiovascular Risk in Diabetes study [99], ONTARGET^®^[33] and TRANSCEND^®^[70] have shown CV and renal benefits with ACE inhibitors or ARBs in patients with T2DM.

In ONTARGET^®^, dual RAS inhibition was not found to be beneficial and was associated with increased harm. The ALiskiren Trial in Type 2 diabetes Using carDio-renal Endpoints (ALTITUDE) study evaluated the addition of aliskiren to an ACE inhibitor or an ARB in T2DM patients with CKD, CVD or both, but was stopped early due to safety concerns [66,100]. The study randomized 8,506 patients to aliskiren or placebo added to standard cardiorenal-protective treatment (an ACE inhibitor or an ARB). The trial was stopped early because, after the seventh interim review of data, the Data Monitoring Committee identified increased incidence of AEs (renal dysfunction, hyperkalaemia, hypotension and an excess of strokes) in patients in the aliskiren arm and concluded that patients were unlikely to benefit from aliskiren treatment added on top of standard antihypertensives [66]. The Combination Angiotensin Receptor Blocker and Angiotensin-Converting Enzyme Inhibitor for Treatment of Diabetic Nephropathy (VA NEPHRON-D) multicentre trial to assess the effect of the combination of losartan and lisinopril compared with losartan alone, on the progression of kidney disease in 1,850 patients with diabetes and overt proteinuria, was terminated recently for similar reasons to those of the ALTITUDE study [61,69].

In the Aliskiren in the Evaluation of Proteinuria in Diabetes (AVOID) study, 599 patients with T2DM, hypertension and nephropathy were randomized to losartan or losartan plus aliskiren. After 6 months’ treatment, the dual RAS inhibition treatment was associated with a 20% reduction in the mean urinary albumin-to-creatinine ratio compared with single RAS inhibition treatment (p < 0.001). The mean rate of decline in eGFR was lower with dual RAS inhibition treatment, but not significantly different from single RAS inhibition treatment [12]. In a small, randomized, crossover trial study in 26 patients with T2DM, hypertension and albuminuria, dual RAS inhibition with aliskiren plus irbesartan was associated with a significantly greater reduction in albuminuria than either of the single RAS inhibition treatments (dual RAS inhibition, –71% reduction; aliskiren, –48%; irbesartan, –58%; p < 0.001 versus aliskiren; p = 0.028 versus irbesartan) [101].

In a recent post-hoc subgroup analysis of patients with diabetes, with or without nephropathy, from the ONTARGET^®^ trial, it was observed that combination therapy with telmisartan plus ramipril did not increase the risk of stroke, other major CV or renal (dialysis or doubling of serum creatinine) outcomes compared with monotherapy, but the AEs of acute dialysis, hyperkalaemia and hypotension occurred more frequently with combination therapy [102]. In the Valsartan Aliskiren Hypertension Diabetes (VIvID) study, 1,143 hypertensive participants with T2DM and stage 1 or 2 CKD were randomized to receive combination aliskiren ⁄ valsartan 150 ⁄ 160 mg or valsartan 160 mg monotherapy for 2 weeks, with force-titration to 300 ⁄ 320 mg and 320 mg, respectively, for another 6 weeks. Combination treatment had additive effects on BP reduction, and tolerability was similar to valsartan [103].

In an open-label, randomized, parallel-controlled study, 64 hypertensive patients with T2DM and CKD, on telmisartan 80 mg plus amlodipine 5 mg treatment, were allocated to receive either aliskiren (150 mg increased to 300 mg) or an increased dose of amlodipine (10 mg). After 24 weeks, there was no significant difference between the two groups in BP decrease, serum creatinine levels or eGFR rate. However, plasma aldosterone levels were significantly decreased in the aliskiren group compared with the amlodipine group, and this decrease correlated significantly with the decrease in urinary 8-hydroxy-2′-deoxyguanosine and liver-type fatty acid binding protein, suggesting that the addition of aliskiren to the maximal recommended dose of ARB and usual dose of amlodipine is more effective in reducing albuminuria and oxidant stress in hypertensive diabetic patients with CKD than increasing the dose of amlodipine [104].

### High-risk patients with a broad range of CV risk factors and TOD

ONTARGET^®^ was one of the largest trials conducted in over 25,000 patients at high CV risk (defined as those with coronary, peripheral or cerebrovascular disease, or diabetes with end-organ damage). The incidence of the primary endpoint (a composite of CV death, MI, stroke or hospitalization for HF) was similar in the dual and two single RAS inhibition groups after a median follow-up of 56 months. However, telmisartan was better tolerated, even in this population that had been screened for tolerance of ACE inhibitors. Compared with ramipril alone, telmisartan plus ramipril was associated with significantly higher incidences of hypotensive symptoms (4.8% versus 1.7%; p < 0.001), syncope (0.3% versus 0.2%; p = 0.03) and renal dysfunction (13.5% versus 10.2%; p < 0.001) [33]. The (AVANT GARDE)-TIMI 43 Trial recruited 1,101 stable patients post acute coronary syndrome having an increase in natriuretic peptides (NP) without heart failure or left ventricular dysfunction < 40%. They were randomized to 8 weeks’ treatment with aliskiren, valsartan, their combination or placebo, and there was no evidence for a benefit of early treatment initiation with valsartan, aliskiren or their combination compared with placebo [105].

A study in patients with MI, the Aliskiren Study in Post-MI patients to Reduce rEmodeling (ASPIRE) study, compared aliskiren with placebo as add on to standard therapy (an ACE inhibitor or ARB and a β-blocker) [67]. At the end of a 36-week treatment period, no difference was observed in the primary endpoint of end-systolic volume change (aliskiren, –4.4 ± 16.8 ml; placebo, –3.5 ± 16.3 ml; p = 0.44). Furthermore, no differences in composite endpoint of CV death, hospitalization for HF or reduction in LVEF > 6 points were observed (aliskiren, 7%; placebo, 6%; p = 0.85). Aliskiren added to standard therapy was associated with more AEs including hypotension, and increases in creatinine and hyperkalaemia. In 17 patients with coronary artery disease (CAD) and SBP >130 mmHg on ACE inhibitor or ARB therapy, addition of aliskiren 150 mg for 6 weeks significantly reduced SBP and plasma renin activity but did not affect plasminogen activator inhibitor-1, fibrinogen, or D-dimer levels, suggesting that dual RAS inhibition does not have any effect on biomarkers of the fibrinolytic system [106].

## Discussion

Chronic RAS inhibition using ACE inhibitors or ARBs as monotherapy is the standard of care to treat the vasoconstrictive / pro-inflammatory effects of RAS activation. Despite a strong biological rationale, dual RAS blockade has been largely disappointing, with initial benefits seen on surrogate endpoints [55] not being translated to meaningful superior clinical benefit [80]. Data from outcome trials such as ONTARGET^®^, VA NEPHRON-D, ALTITUDE and ASTRONAUT have shown no renal or CV outcome benefits with dual RAS inhibition, and have even shown deleterious effects across most patient groups. For ACE inhibitor / ARB dual therapy, benefits appear confined to symptomatic HF in patients whose symptoms persist in the presence of maximized ACE inhibitor and beta-blocker treatment and not immediately post-MI, and arguably in severe hypertension while balancing the potential adverse effects [107].

The ONTARGET^®^ trial was the first to allude to the need for caution over the use of dual RAS inhibition, even though the study was not designed to measure the impact of treatment on renal events [33]. A systematic review of ARBs and ACE inhibitors in ischaemic heart disease concluded that dual RAS inhibition with an ARB and ACE inhibitor is no better than ACE inhibitor or ARB therapy alone and increases the risk of harm [108]. Dual RAS blockade was not beneficial for clinically relevant endpoints in patients with diabetic nephropathy [109]. A systematic review of randomized controlled trials reported until 2009 also showed lack of evidence to support the use of combination therapy in people with albuminuria and one or more CV risk factors [110]. It is of interest that small studies have shown some benefits in the reduction of proteinuria with dual RAS blockers in primary glomerulopathy [82,85].

Dual RAS inhibition studies involving aliskiren also have not shown consistent benefits but have reported increased harm with combination treatment. In patients with uncomplicated Type 1 diabetes, dual RAS blockade with aliskiren and ramipril was associated with greater arterial compliance, flow-mediated vasodilatation and renal vasodilatation [111]. The AVOID and ALTITUDE studies conducted in patients with diabetes and advanced CKD did not show beneficial effects of dual RAS inhibition involving aliskiren, while in the VIvID study conducted in patients with diabetes and early stages of CKD, the tolerability of combination therapy with aliskiren and valsartan was similar to valsartan monotherapy, although the study was of a short duration. A meta-analysis that assessed the combination of aliskiren with other RAS inhibitors reported an increased risk of hyperkalaemia (but not acute kidney injury) with combination treatment [112]. A post-hoc subgroup analysis of ONTARGET^®^ showed no increase in the risk of stroke, other major CV or renal outcomes in patients with diabetes with or without nephropathy [102], unlike the ALTITUDE trial results which suggested that use of aliskiren in combination with an ACE inhibitor or an ARB increased risk of stroke in people with diabetes [100]. However, in both studies, there was an increase in adverse renal events, suggesting dual RAS inhibition is not beneficial in these patients [102]. The VA NEPHRON-D trial was terminated recently for similar reasons to those of ALTITUDE [69,113]. A recent meta-analysis of 9 studies with 11,543 patients with diabetes showed that patients with diabetes receiving dual RAS blockade had a higher risk (versus monotherapy) of hyperkalaemia, hypotension and kidney damage, with no reduction in overall mortality. Except for kidney damage, removing the most influential study (ALTITUDE) from the analysis did not substantially alter the results of the analyses [114]. Hence, recent evidence suggests that these combinations should not be routinely prescribed in patients with diabetes until further data become available from other future studies [115].

Despite the lack of outcome evidence, dual RAS inhibition was recommended in several guidelines on the basis of changes in surrogate endpoints such as BP, proteinuria and endothelial dysfunction [116]. However, the clinical outcomes and safety data have not confirmed indications to dual RAS blockade in essential hypertension treatment, HF and ischaemic heart disease [117]. Existing evidence indicates that it is reasonable for continued use of ACE inhibitor plus ARB combination for proteinuric nephropathies and resistant hypertension [117], and for diabetic nephropathy [107]. Ongoing studies that may give further insight into the role of dual RAS inhibition are the Long-term Impact of RAS Inhibition on Cardiorenal Outcomes (LIRICO), and Aliskiren Trial of Minimizing OutcomeS for Patients with HEart failure (ATMOSPHERE) comparing aliskiren with aliskiren plus enalapril in patients with chronic HF. Multi-agent, low-level intervention earlier in the disease process is yet to be explored [107].

The Canadian Hypertension Education Program has recommended against use of ARBs and ACE inhibitors together except in patients with HF refractory to ACE inhibitor [118]. The American Heart Association guidelines currently do not recommend the use of ACE inhibitors and ARBs together, but there is no specific directive warning against their use in combination. The United States Food and Drug Administration also has not issued any warnings. In 2012, the National Healthcare Services professionals were advised to initiate combination of an ACE inhibitor and ARB only under specialist supervision in patients with renal disease, taking into account the potential for adverse effects such as hyperkalaemia [119]. Based on the ALTITUDE study results, dual aliskiren with ACE inhibitor/ARB therapy is now contraindicated by the US Food and Drug Administration in patients with diabetes and is to be avoided in patients with moderate renal impairment (GFR < 60 ml/min) [120], and is contraindicated by the European Medicines Agency (EMA) in patients with diabetes and in patients with moderate renal impairment (GFR < 60 ml/min) [121].

A recent systematic review and meta-analysis of all randomized controlled trials reported between January 1990 and August 2012 comparing dual blockers of the RAS with monotherapy, and reporting data on either long-term efficacy (≥ 1 year) or safety events (≥ 4 weeks), and with a sample size of at least 50 (68,405 patients [mean age 61 years, 71% men] and mean duration of 52 weeks) showed that although dual blockade of the RAS had seemingly beneficial effects on certain surrogate endpoints, it failed to reduce mortality and was associated with an excessive risk of AEs such as hyperkalaemia, hypotension and renal failure compared with monotherapy. The results of this most comprehensive meta-analysis to date indicated the risk-benefit ratio argues against the use of dual therapy [113]. Based on these study results, the EMA recently started a review of the risks of combining certain medicines to block separate stages of the RAS in the treatment of hypertension and congestive heart failure [122].

Selection of the most appropriate antihypertensive combination should be dependent on careful review of the individual patient and appropriate consideration of drug pharmacology. Existing evidence suggests that in CV high-risk patients and those with evidence of renal disease, the use of an ARB plus calcium channel blocker is the preferred combination due to superior renoprotective and CV benefits and reduced metabolic side effects in patients with concomitant metabolic disorders [123].

## Conclusions

Although some small studies demonstrate benefits of combinations of ARBs with ACE inhibitors, larger clinical trials such as ONTARGET^®^ and VA NEPHRON-D indicate that this approach to dual RAS inhibition does not improve outcomes across most patient groups, and in fact, increases the risk of AEs. Similarly, dual RAS inhibition involving aliskiren has been reported in large trials such as ALTITUDE and ASTRONAUT to increase the risk of AEs with no clinical benefits. A recent meta-analysis of all major trials involving dual RAS inhibition has clearly shown that the risk-benefit ratio argues against the use of dual RAS inhibition. Based on these results, the EMA currently is reviewing the use of dual RAS blockade in the treatment of hypertension and congestive heart failure.

## Abbreviations

ACE: Angiotensin-Converting Enzyme; AE: Adverse Event; ALLAY: ALiskiren Left ventricular Assessment of HypertrophY; ALOFT: ALiskiren Observation of heart Failure Treatment; ALTITUDE: Aliskiren Trial in Type 2 Diabetes Using Cardio-Renal Endpoints; ARB: Angiotensin II Receptor Blocker; ASPIRE: Aliskiren Study in Post-MI patients to Reduce rEmodeling; ASTRONAUT: AliSkiren TRial ON Acute heart failure oUTcomes; AT1: Angiotensin II Type 1 receptor; AT2: Angiotensin II Type 2 receptor; ATMOSPHERE: Aliskiren Trial of Minimizing OutcomeS for Patients with HEart failuRE; (AVANT GARDE)-TIMI 43: Aliskiren and Valsartan to Reduce NT-proB-type natriuretic peptide (BNP) via Renin-Angiotensin-Aldosterone-System Blockade; AVOID: Aliskiren in the eValuation of prOteinuria in Diabetes; BP: Blood Pressure; CHARM: Candesartan in Heart failure Assessment of Reduction in Mortality and Morbidity; CHD: Coronary Heart Disease; CI: Confidence Interval; CKD: Chronic Kidney Disease; COOPERATE: Combination Treatment of angiotensin II receptor blocker and Angiotensin-Converting Enzyme inhibitor in nondiabetic renal Disease; CV: Cardiovascular; CVD: Cardiovascular Disease; DRI: Direct Renin Inhibitor; ELITE: Evaluation of Losartan In The Elderly; EMA: European Medicines Agency; ESRD: End-Stage Renal Disease; eGFR: estimated Glomerular Filtration Rate; HF: Heart Failure; IDDM: Insulin-Dependent Diabetes Mellitus; IgA: Immunoglobulin A; LIRICO: Long-term Impact of RAS Inhibition on Cardiorenal Outcomes; LVD: Left Ventricular Dysfunction; LVEF: Left Ventricular Ejection Fraction; LVH: Left Ventricular Hypertrophy; MI: Myocardial Infarction; NT-proBNP: N-terminal Prohormone of Brain Natriuretic Peptide; NO: Nitric Oxide; ONTARGET®: ONgoing Telmisartan Alone and in combination with Ramipril Global Endpoint Trial; PRA: Plasma Renin Activity; PRC: Plasma Renin Concentration; PPAR-γ: Peroxisome Proliferator-Activated Receptor Gamma; RAS: Renin Angiotensin System; RR: Risk Reduction; SNS: Sympathetic Nervous System; T2DM: Type 2 diabetes mellitus; TOD: Target Organ Damage; tPA: tissue Plasminogen Activator; TRANSCEND®: Telmisartan Randomized AssessmeNt Study in ACE-I iNtolerant subjects with cardiovascular Disease; UACR: Urinary Albumin-Creatinine Ratio; Val-HeFT: Valsartan in Heart Failure Trial; VALIANT: VALsartan in Acute myocardial iNfarcTion; VA NEPHRON-D: Combination Angiotensin Receptor Blocker and Angiotensin-Converting Enzyme Inhibitor for Treatment of Diabetic Nephropathy; VIvID: Valsartan Aliskiren Hypertension Diabetes.

## Competing interests

The author declares that he has no competing interests.

## Author’s contributions

SM was fully responsible for all content and editorial decisions, was involved at all stages of the manuscript (including concept development and critical review and revision of the article), and has approved the final version of the review that reflects the author’s interpretation and conclusions.

## Author’s information

SM is Associate Professor of Clinical Medicine and Director of the End-Stage Renal Disease Program in the Division of Nephrology and Hypertension, Department of Internal Medicine, at the American University of Beirut Medical Center.
